# Prophage Carriage and Genetic Diversity within Environmental Isolates of *Clostridioides difficile*

**DOI:** 10.3390/ijms25010002

**Published:** 2023-12-19

**Authors:** Khald Blau, Claudia Gallert

**Affiliations:** Department of Microbiology–Biotechnology, Faculty of Technology, University of Applied Sciences Emden/Leer, 26723 Emden, Germany; khald.blau@hs-emden-leer.de

**Keywords:** *Clostridioides difficile*, prophages, CRISPR-Cas systems, phage-related genes, sequence types, ribotypes, intact prophages

## Abstract

*Clostridioides difficile* is an important human pathogen causing antibiotic-associated diarrhoea worldwide. Besides using antibiotics for treatment, the interest in bacteriophages as an alternative therapeutic option has increased. Prophage abundance and genetic diversity are well-documented in clinical strains, but the carriage of prophages in environmental strains of *C. difficile* has not yet been explored. Thus, the prevalence and genetic diversity of integrated prophages in the genomes of 166 environmental *C. difficile* isolates were identified. In addition, the clustered regularly interspaced short palindromic repeats (CRISPR)-Cas systems were determined in the genomes of prophage regions. Predicted prophages and CRISPR-Cas systems were identified by using the PHASTER web server and CRISPRCasFinder, respectively. Phylogenetic relationships among predicated prophages were also constructed based on phage-related genes, terminase large (TerL) subunits and LysM. Among 372 intact prophages, the predominant prophages were phiCDHM1, phiCDHM19, phiMMP01, phiCD506, phiCD27, phiCD211, phiMMP03, and phiC2, followed by phiMMP02, phiCDKM9, phiCD6356, phiCDKM15, and phiCD505. Two newly discovered siphoviruses, phiSM101- and phivB_CpeS-CP51-like *Clostridium* phages, were identified in two *C. difficile* genomes. Most prophages were found in sequence types (STs) ST11, ST3, ST8, ST109, and ST2, followed by ST6, ST17, ST4, ST5, ST44, and ST58. An obvious correlation was found between prophage types and STs/ribotypes. Most predicated prophages carry CRISPR arrays. Some prophages carry several gene products, such as accessory gene regulator (Agr), putative spore protease, and abortive infection (Abi) systems. This study shows that prophage carriage, along with genetic diversity and their CRISPR arrays, may play a role in the biology, lifestyle, and fitness of their host strains.

## 1. Introduction

*Clostridium difficile*, recently reclassified as *Clostridioides difficile*, is a Gram-positive, spore-forming, anaerobic bacterium that can cause *C. difficile* infection (CDI), and leading to mild to severe diarrhoea, pseudomembranous colitis and toxic megacolon [1,2,3]. CDI typically occurs after the use of antibiotics, which destabilise the commensal gut microbiota and allow the vegetative cells and spores of *C. difficile* to proliferate [4].

*C. difficile* bacteriophages, also known as *C. difficile* phages, are viruses that infect and kill vegetative *C. difficile* cells. Bacteriophages are, therefore, important drivers for the evolution and biology of bacterial pathogens [5]. *C. difficile* phages are classified in either the *Myoviridae* or *Siphoviridae* families of the order *Caudovirales*. *Myoviridae* is the common family of *C. difficile* phages, which have a long and nonflexible tail tube surrounded by a contractile tail sheath [6,7]. Phages of the *Siphoviridae* family have long, flexible, and non-contractile tails [8]. The natural resistance of *C. difficile* to a wide range of antibiotics has led researchers to investigate the use of phage therapy to treat CDI [9,10,11,12]. However, prophage presence or absence and impact on *C. difficile* biology and lifestyle is still poorly understood due to a lack of knowledge about the genetic background of the host strains. To provide a basis for the development of phage therapy for CDI, it is necessary to understand the biology and genetics of *C. difficile* bacteriophages/prophages.

At least 11% of the genome of the *C. difficile* reference strain CD630 is composed of mobile genetic elements (MGEs), including prophages [13]. Therefore, the analysis of prophages, which typically integrate into the host genome, is an important step prior to the induction, genetic characterisation, and sequencing of novel phages. Putative prophage regions have been identified in several *C. difficile* ribotypes (RTs) and toxin types, including RT012, RT027, RT078, and RT106, as well as toxigenic and nontoxigenic strains [9]. Variation in RTs within a group could contribute to different patterns of prophage carriage. A likely reason for this limited data and the small number of fully characterised phages is the lack of suitable bacterial hosts and conditions for isolating and propagating these phages.

Prophages in the genomes of *C. difficile* strains can have a wide range of effects on the virulence, biology, and evolution of their host strains. The number of studies investigating prophage-mediated gene regulation in *C. difficile* is limited. Phage transduction of antimicrobial resistance has recently been reported in *C. difficile* strains. For instance, phiC2 mediated the transduction of Tn*6215*, encoding erythromycin resistance, between *C. difficile* isolates [14]. Two other studies reported that the prophages phiCD119 and phiCD38-2 could repress pathogenicity locus (PaLoc) genes and stimulate toxin production in *C. difficile* lysogens [15,16] or carry *agr*-like quorum sensing (QS) systems, which regulate multiple virulence factors [17]. Recently, Graneau et al. [18] reported that the large phiCD211 prophages in *C. difficile* possess several phage-unrelated genes, including AcrB/AcrD/AcrF multidrug resistance proteins, EzrA septation ring formation regulator, and putative YyaC-like spore germination protease, which can potentially affect host lifestyle and biology. In addition, the phage-related PaLoc can be transferred via transduction between *C. difficile* strains, which converts nontoxigenic strains into toxin producers [19].

Although the reason for the lack of isolation of strictly virulent phages remains unknown, it may be largely related to adaptation strategies in which *C. difficile* prophages have evolved to coexist with their host genomes through lysogeny to enhance survival under harsh environmental conditions [20]. The specificity of the phage–host interactions depends on the mechanisms that the bacteria use to resist infection by the phage. In this context, all characterised genomes of most sequenced *C. difficile* carry multiple clustered regularly interspaced short palindromic repeats (CRISPR), and the phages themselves encode CRISPR arrays, targeting corresponding and/or additional phages [20] and the spread of CRISPR arrays through horizontal gene transfer (HGT) by lysogenic phages [21], could be observed. However, the abundance and diversity of prophages in clinical strains of *C. difficile* have been thoroughly documented. Nevertheless, the presence of prophages in environmental strains of *C. difficile* in diverse environmental sources, such as wastewater treatment plants, faeces of claves, soil, biogas plants, and thermophilic digesters for treating sewage sludge or biowaste remains unexplored and also the potential risk.

In this study, the whole genomes of 166 environmental *C. difficile* isolates from diverse environmental samples were sequenced and annotated as previously described [22] and used as a source for exploiting the prevalence and genetic diversity of integrated prophages in their genomes. Additionally, the predicted prophages could be correlated with the well-described sequence types (STs) and RTs of those environmental *C. difficile* strains. The identified prophage genomes were analysed to determine their overall genetic relatedness based on some phage-related gene products, such as terminase large subunits (TerL) and LysM proteins. The genomes of two newly identified phiSM101- and phivB_CpeS-CP51-like *Clostridium* phages were characterised. Furthermore, the CRISPR-Cas systems in the genomes of prophage regions were determined.

## 2. Results

### 2.1. Prevalence of Prophages in the Genomes of Environmental C. difficile Isolates

**Figure 1:**
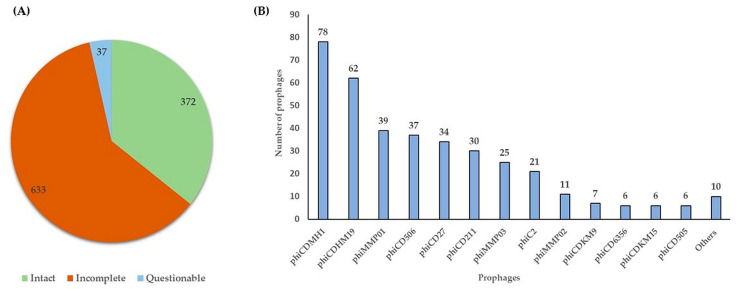
Prophage analysis of 166 environmental *C. difficile* isolates. (**A**) predicated prophages and (**B**) the predominant intact prophages identified in *C. difficile* genomes. “Others” indicate isolate genomes with fewer than three assigned intact prophages.

Prophages were predicated in varying numbers in all genomes of 166 environmental *C. difficile* isolates tested, using PHASTER (PHAge Search Tool–Enhanced Release) web server. The prophage sequences were categorised into intact, incomplete, and questionable based on score values of >90, <70, and 70–90, respectively [23]. In total, 372, 633, and 37 intact, incomplete, and questionable prophages, respectively, were predicated (Figure 1A, Table S1). One, two and ten isolates contained the highest numbers of predicated prophages (12, 11, and 10 prophages, respectively), while other isolates hosted between four and nine prophages (Table S1). A total of 20 different intact prophages were identified, ranging between one and six per isolate. Among 372 intact prophages, the predominant prophages were phiCDHM1 [78, (21%)], phiCDHM19 [62, (17%)], phiMMP01 [39, (10%)], phiCD506 [37, (10%)], phiCD27 [34, (9%)], phiCD211 [30, (8%)], phiMMP03 [25, (7%)], phiC2 [21, (6%)], and phiMMP02 [11, (3%)], followed by phiCDKM9 [7, (2%)], phiCD6356, phiCDKM15, and phiCD505 [6, (2%), each) (Figure 1B). The remaining intact prophages were presented by one or two isolates, including phiSM101- and phivB_CpeS-CP51-like *Clostridium* phages, phiCD111, phiMMPO4, phiCD38-2, phiCDHM14, and phi0305phi8_36-like *Bacillus* phage. Most of these prophages belonged to the *Myoviridae* family, whereas six different prophages belonged to the *Siphoviridae* family of the order *Caudovirales*, as previously described [20].

Most of identified prophages were homologous to known phages reported previously in *C. difficile*, although a few were similar to other bacterial phages, such as phiSM101- and phivB_CpeS-CP51-like *Clostridium* phages, which were first discovered in *C. perfringens* strains [24] and phi0305phi8_36-like *Bacillus* phage, which belongs to *Bacillus* phages [25] (Table S1). To the best of our knowledge, this is the first study which identifies two newly discovered prophages, phiSM101- and phivB_CpeS-CP51-like *Clostridium* phages, in the genomes of environmental *C. difficile* isolates (RSS7 and DS169, respectively), which are similar to other predicated prophages, localised on the genomes of *C. difficile* W0003a and NT64 reference strains (GenBank accession no. CP101707.1 and CP025047.1, respectively). These integrated prophages had not previously been identified in genomes of *C. difficile* W0003a and NT64 strains. 

In the present study, extrachromosomal circle contigs were detected after whole genome sequencing (WGS), assembly, and annotation of *C. difficile* isolates. The prophages phiCD211, phiCD38-2, phiCD506, phiCD6356, phiCD111, and phiCDHM14 are maintained as extrachromosomal (independent) plasmids in lysogens, despite encoding predicated integrases within their genomes as previously reported [9]. Their genome size ranged from 45 to 145 kb, 36 to 37 kb, 32 to 34 kb, 35 to 37 kb, ~39 kb, and ~36 kb, respectively (Table S1).

### 2.2. Prevalence and Diversity of Intact Prophages in STs/RTs of Environmental C. difficile Isolates 

**Figure 2:**
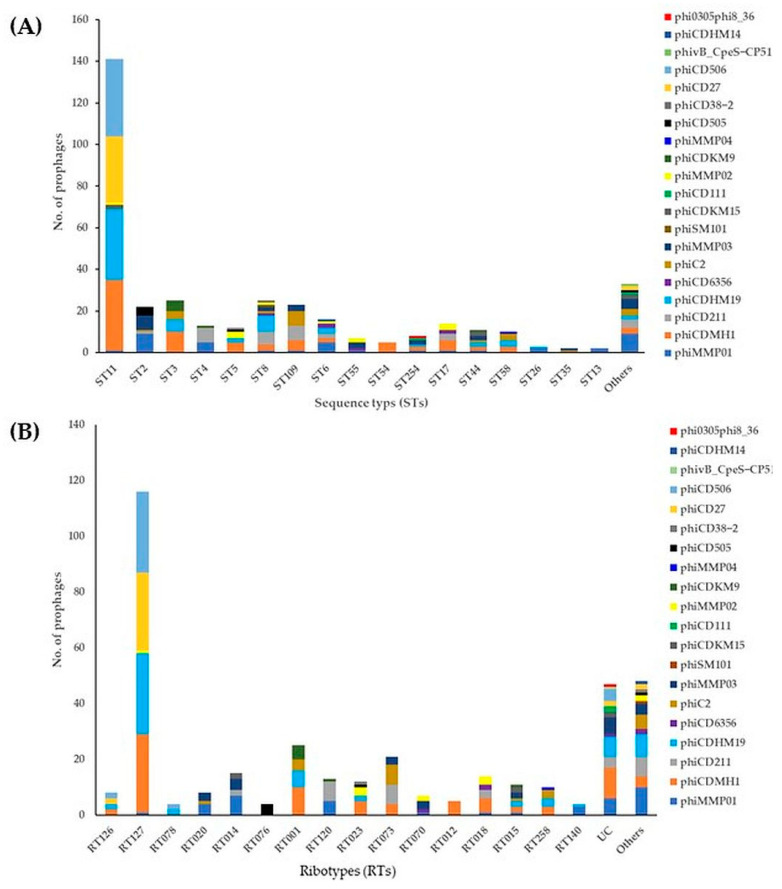
Prevalence of intact prophages in the genomes of environmental *C. difficile* isolates (*n* = 166). (**A**) Sequence types; (**B**) Ribotypes. UC: unclassified RTs. Others indicate intact prophages with fewer than three assigned ST/RT strains.

Most of the 372 intact prophages were found within ST11 [141, (38%)], ST3 and ST8 [25, (7%), each], ST109 [23, (6%)], and ST2 [22, (6%)], followed by ST6 [16, (4%)], ST17 [14, (4%)], ST4 [13, (3%)], ST5 [12, (3%)], ST44 [11, (3%)], and ST58 [10, (3%)] (Figure 2A, Table S2). The most predominant prophages localised on the genomes of ST11 strains from clade 5 were phiCD506, phiCDHM1, phiCDHM19, and phiCD27. Among these STs, ST8 showed the greatest diversity (10 different prophages), ST11, ST6, and ST44 (7 prophages, each), ST254 (6 prophages), ST2, ST5, ST109, ST1074, and ST17 (5 prophages, each), and ST3, ST55, ST58, ST49, ST821, and ST53 (4 prophages, each). One to three different prophages were found in the remaining STs summarised as “others” (Figure 2A, Table S2). In addition, phiCD27 was identified only in ST11 and ST15/ST53 strains with 32 and 1 prophage, respectively, while phiCD506 was identified only in ST11 strains from clade 5. Of the intact prophages, phiMMP01 had the highest diversity with 20 distinct STs, phiCDHM1 and phiMMP03 (12 STs, each), phiCD211 and phiC2 (10 STs, each), and phiCDHM19 (11 STs). The remaining intact prophages had between one and six different STs (Figure 2A, Table S2).

Among 372 intact prophages, the most prophages were identified in RT strains, including RT127 [116, (31%)], RT001 [25, (7%)], RT073 [21, (6%)], RT014 [15, (4%)], and RT018 [14, (4%)], followed by RT120 [13, (3.4%)], RT023 [12, (3%)], RT015 [11, (3%)], and RT258 [10, (3%)] (Figure 2B and Table S3). The most common prophages located in the genomes of RT127 strains were phiCD506, phiCDHM1, phiCDHM19, and phiCD27. The RT isolates contained a varying number of intact prophages: RT015 (seven different prophages), RT127 (six prophages), RT023 (five prophages), and RTs 126, 014, 001, 073, 070, and 258 (four prophages each), demonstrating significant diversity of prophage carriage within the same RT (Figure 2B and Table S3). An obvious correlation could be found between the presence of intact prophage types and specific STs/RTs; for instance, the phiCD506 was identified only in RT127/ST11 strains from clade 5 (Figure 2 and Table S3). Interestingly, three newly discovered prophages, namely phiSM101- and phivB_CpeS-CP51-like *Clostridium* phages and phi0305phi8_36-like *Bacillus* phage, could be identified in RSS7 (RT159/ST8), DS169 (RTUC/ST821), and DSS202 (RTUC/ST258) isolates, respectively (Table S1).

### 2.3. Phylogenetic Analysis of Terminase Large Subunits (TerL) and LysM Proteins of C. difficile Prophages

**Figure 3:**
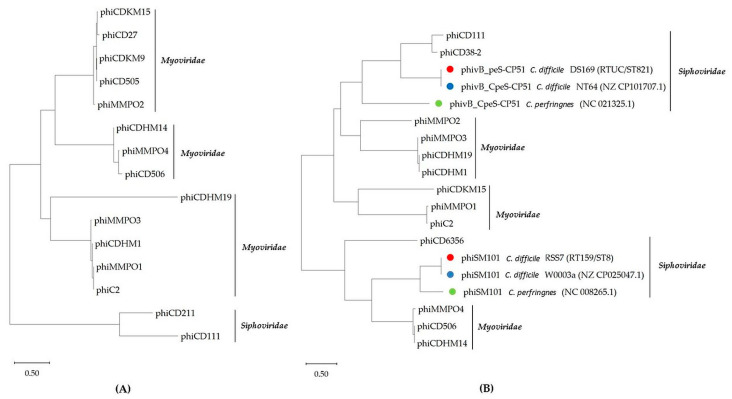
Maximum likelihood phylogenetic analysis of *C. difficile* prophage LysM proteins (**A**) and terminase large subunits (TerL) (**B**). Reference strains, *C. difficile* NT64/W0003a (blue circle) and reference phages of *C. perfringens* phivB_CpeS-CP51/phiSM101 (green circle) were used to compare them with newly predicted prophages, phiSM101- and phivB_CpeS-CP51-like *Clostridium* phages in the genomes of *C. difficile* DS196 and RSS7 strains (red circle).

A phylogenetic tree was generated for the amino acid sequence of TerL or LysM proteins, depending on the presence of these genes in all identified intact prophages of environmental *C. difficile* isolates (Figure 3). The phi0305phi8_36-like *Bacillus* phage, belonging to *Bacillus* phages, was excluded from the phylogenetic analysis. This prophage does not contain either LysM or TerL proteins. This newly discovered prophage can be named the “cryptic prophage” as it has lost critical genes for infecting and generating phage progeny. The LysM domain was first identified in bacteriophage lysin proteins that hydrolysed the peptidoglycan component of the bacterial cell wall. The amino acid sequences of the LysM proteins from each representative intact prophage were used to generate the phylogenetic tree (Figure 3A). Maximum likelihood (ML) analysis was applied to LysM sequences from 15 representative intact prophages. The resulting tree showed the taxonomic separation between siphoviruses and myoviruses, with taxa grouped into clades. The myoviruses consist of 13 taxa, while the siphoviruses clade comprises two taxa (Figure 3A). Phylogenetic analysis of the LysM sequences identified four groups of *C. difficile* prophages, three within the myoviruses and one within the siphoviruses group.

To determine the phylogenetic relationships among the TerL proteins of representative complete prophages, including the newly identified phiSM101- and phivB_CpeS-CP51-like *Clostridium* phages, implying that prophages with closely related TerL proteins display analogous DNA packaging strategies (Figure 3B). ML analysis was conducted on TerL sequences from 15 representative intact prophages, resulting in a tree that demonstrated the taxonomic division between siphoviruses and myoviruses, with taxa clustered into clades. The myovirus group comprised 10 taxa, while the siphovirus group included five taxa. Using TerL sequences, the phylogenetic analysis identified five clusters of *C. difficile* prophages, with three belonging to the myovirus group and two to the siphovirus group (Figure 3B). The siphoviruses, phivB_CpeS-CP51- and phiSM101-like *Clostridium* phages showed clustering, with sub-clusters observed for the phages that split into a group containing prophages identified in DS169 (RTUC/ST821) and NT64 (reference) strains for phivB_CpeS-CP51-like *Clostridium* phages, as well as RSS7 (RT159/ST8) and W0003a (reference) strains for phiSM101-like *Clostridium* phages, and two singletons for the reference *C. perfringens* phages, phivB_CpeS-CP51 and phiSM101.

Prophages, phiCDHM14, phiMMPO4, and phiCD506 were clustered together based on TerL sequences, in agreement with the clustering based on the sequence of LysM proteins (Figure 3). In contrast, the phiCDHM19 phage forms a clade with phiCDHM1 and phiMMP03 (Figure 3B), which is incompatible with the LysM analysis (Figure 3A), hence proving that these phages with closely related TerL proteins share comparable DNA packaging strategies. In another case, the prophages phiMMP03, phiCDHM1, phiMMP01, and phiC2 were clustered in the same clade due to their LysM protein sequences, which is inconsistent with the results of the TerL sequence analysis. It illustrates that these prophages may have identical host cell lysis by sharing the same evolutionary history of the phage genomes but differing DNA packaging strategies.

### 2.4. Phage-Unrelated Genes of Intact Prophages Identified in C. difficile Genomes

Several phage genomes contain “cargo genes” that are not related to the phage replication cycle. Their expression is frequently independent of the phage cycle and occurs during lysogeny. These gene products potentially affect the lifestyle and fitness of their hosts. In this study, the genomes of environmental *C. difficile* prophages encode genes that are likely to have an effect on their hosts. For instance, 5% of the phiCDHM1 prophages encode homologous accessory gene regulator (Agr) quorum sensing (QS) system controls virulence factor expression, namely *agrD* (a pre-peptide of autoinducing peptide, AIP), *agrB* (processing of the pre-AIP), and *agrC* (a histidine kinase that activates the response regulator), whereas QS system, including *agrD* and *agrC*, were detected in phiMMP01, phiMMP02, and phiMMP03 prophages [(1/39, 3%), (1/11, 9%), and (1/25, 4%), respectively] (Table 1). Although no *agrA*-like response regulator was identified in the phiCDHM1, the function of these genes in QS in *C. difficile* requires further investigation. In addition, resistance strategies, including abortive infection (Abi) systems, were identified in phiMMP01 and phiC2 prophages [(19/39, 49%) and (6/21, 29%), respectively], that promote cell death and block phage multiplication within a bacterial population.

Several genes were identified in phiCD211 that possibly influence bacterial biology and lifestyle, including the death-on-curing (DOC) family and the spore protease YyaC. The DOC family protein was identified in phiCD211 (15/30, 50%), providing further evidence for the episomal nature of phiCD211, and loss of the prophage should lead to host death. The spore protease YyaC was also identified in 19 out of 30 phiCD211 prophages (Table 1); the presence of YyaC in phiCD211 could have an effect on the sporulation and/or germination of its host strains. Additionally, the phiCD211 genomes contain a tRNA for serine (Ser-GCT, 22/30; 73.3%) and serine/isoleucine (IIe-TAT/Ser-GCT, 4/30; 13.3%) (Table S1). The majority of the phiCD211 genomes also encode DNA methyltransferases and anti-repressor proteins, which may be involved in protecting the phage from host defence mechanisms. Overall, the phiCD211 genome contained noteworthy features, including multiple gene products that may affect the physiology and fitness of its hosts. The presence of partition genes, such as *parA*, was identified in some of the prophages phiCDHM14 and phiCD6356. These prophages reside as extrachromosomal plasmids within the genomes of *C. difficile* (Table 1).

### 2.5. The Genome Features of Newly Discovered phivB_CpeS-CP51- and phiSM101- Clostridium Phages Identified in C. difficile Genomes

The genomes of newly discovered phivB_CpeS-CP51- and phiSM101-like *Clostridium* phages are 30,765 bp and 41,548 bp in length, respectively. Both phages have a G+C content of 28% and 26.3%, respectively (Table S1), which is similar to that of published *C. perfringens* phage genomes with accession numbers NC_021325.1 (phivB_CpeS-CP51) and NC_008265.1 (phiSM101). The identified regions of the newly discovered prophages, which are localised on the host genomes of *C. difficile* RSS7 and DS169 strains using the PHASTER web server, were BLASTn in the NCBI database. The newly discovered phivB_CpeS-CP51- and phiSM101-like *Clostridium* phages are identical to sequence regions in the genomes of *C. difficile* NT64 and W0003a strains with coverage (100 and 99%) and identity (98.7 and 99.8%), respectively. These prophages integrated into the genomes of *C. difficile* W0003a and NT64 strains had not been identified previously. The sequence of phivB_CpeS-CP51-like *Clostridium* phage was found in the genome of strain CD169 from nt 2,180,576 to 2,211,340. The prophage is integrated between unfunctional assigned genes (hypothetical proteins). The sequence of phiSM101-like *Clostridium* phage was found in the genome of strain RSS7 from nt 2,200,495 to 2,242,042. This prophage is integrated into the host genome between the gene encoding for lactate utilisation domain (LUD) protein and ABC transporter ATP-binding protein.

**Figure 4:**
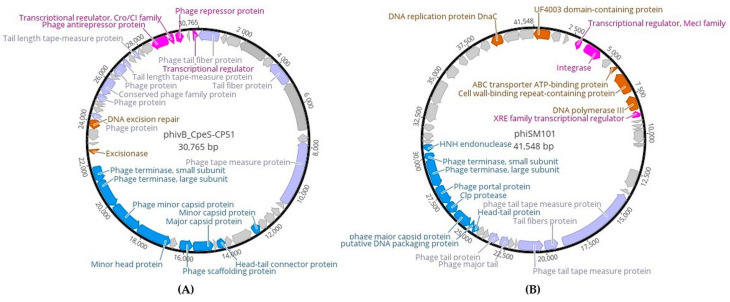
Genome organisation of phivB_CpeS-CP51-like *Clostridium* prophage (**A**) and phiSM101-like *Clostridium* prophage (**B**) assembled as a circle. Predicted CDSs are marked with arrows and colours that indicate functional assignments: packaging and head morphogenesis (blue), DNA replication (brown), tail structural components and assembly (purple), and lysogeny (pink). CDSs with no assigned function are light grey.

In total, the genome of phivB_CpeS-CP51-like *Clostridium* phage had 47 predicated coding DNA sequences (CDSs). No *rRNA-* and *tRNA*-genes were identified. Of these 47 CDSs, 23 (49%) genes were assigned a predicted function, and 24 (51%) were encoded for genes with an unknown function (Figure 4A).

The genome of phiSM101-like *Clostridium* phage had 47 predicated CDSs. Twenty-one (45%) could be assigned for putative functions, and 26 (55%) could not be assigned (Figure 4B). The complete genomes of phivB_CpeS-CP51- and phiSM101-like *Clostridium* phages could be divided into functional clusters that encode predicted proteins involved in packaging and head morphogenesis, tail assembly and structure, DNA replication, transcription, and recombination, and lysogeny control. In addition, predicated protein genes were detected in both prophages, including an integrase, repressor proteins, anti-repressor, excisionase, endonuclease, and putative transcriptional regulators, suggesting that the newly discovered prophages in *C. difficile* genomes could be affected some bacterial functions in their hosts (Figure 4). The gene cluster for host cell lysis, such as putative holin protein, *N*-acetylmuramoyl-L-alanine amidase, and LysM proteins, could not be identified in both prophage genomes, indicating that these prophages could be unable to infect other bacterial strains. The recently discovered prophages could be designated as “cryptic or defective prophages,” having lost crucial genes for infection and producing phage progeny. 

During analysis, genes of phiSM101-like *Clostridium* phage genome involved in DNA replication encoding DnaC protein, ABC transporter ATP-binding proteins, cell wall-binding protein, and DNA polymerase III (Figure 4B) could be detected, whereas phivB_CpeS-CP51-like *Clostridium* phage genome involved extra genes for DNA excision repair and excisionase (Figure 4A). Interestingly, there are no CRISPR arrays and *cas* genes in the genome of both detected prophages. These findings suggest that the prophages carry no spacers and may be unable to provide functional immunity against the corresponding phages.

### 2.6. The CRISPR Arrays and Cas-Systems in Prophages Identified in C. difficile Genomes

As the discovery of CRISPR arrays and Cas systems in prophage regions within environmental *C. difficile* genomes is atypical, their prevalence and diversity were investigated among 1042 identified prophages (Figure 1A), whether they were intact, incomplete, or questionable, using CRISPRCasFinder [26]. A total of 505 CRISPR arrays consisting of various direct repeat (DR) sequences belonged to different families and separated by unique spacers were identified on the genomes of predicted prophages of 165 environmental isolates; however, the *C. difficile* RSS10 strain harboured prophages without homologous CRISPR sequences (Table S4). Additionally, 313 multiple CRISPR arrays were predicated in the genomes of 372 intact prophages; for instance, the prophages phiMMP01 and phiC2 containing CRISPR arrays of two to four, with the number of spacers varying between two and 19 (Table 2 and Table S4). It is worth noting that DR sequences occur in different copies, and all the DRs belong to different families. The DR sequences and their families were correlated with ST/RT and prophage types (Table 2 and Table S4). It was discovered that numerous prophages shared the same DR sequences and families, such as R1411, R7326, and R7360 (Table 2), implying variation in ST/RT. In general, the prophages carry multiple predicted CRISPR arrays (*n* = 1 to 4), with the number of spacers varying between one and 44, and their length ranged from 87 to 2930 bp (Table 2 and Table S4). Interestingly, three newly predicated prophages, namely phivB_CpeS-CP51- and phiSM101-like *Clostridium* phages, and phi0305phi8_36-like *Bacillus* phage, were identified without any CRISPR sequences or Cas types (Table S4). 

Most spacer sequences are unique and can be found in multiple array types and different locations within the CRISPR arrays that carry them. This finding indicates the potential immunity conferred by these prophages-carried spacers across different phage lineages. At least one array with a conserved DR sequence is presented across the prophages, and all the DRs belong to the same family that is also found in *C. difficile* chromosomal arrays (Table 2 and Table S4). The vast majority of prophage genomes were found to be devoid of *cas* genes, and it is assumed that the bacterial Cas proteins are responsible for their processing. Nonetheless, *cas* genes were observed in six prophage genomes. The incomplete and questionable prophages harbouring the TypeI *cas3a* genes were found to be presented in the genomes of strains RS39, CF76, RS147, and RS150, while the Type-IIIA *csm2* gene was identified within phiCDHM14 in the genomes of strain ARC182 (Table S5). No CRISPR arrays or Cas systems were detected in the intact prophages of the “hypervirulent” *C. difficile* RT078 strains; however, they carried only CRISPR arrays in their incomplete prophages (Table S4).

## 3. Discussion

The sequencing and annotation of 166 environmental *C. difficile* genomes have facilitated the identification of numerous putative prophage genomes by using the PHASTER web server. The abundance and genetic diversity of prophages in *C. difficile* strains found in clinical settings have been extensively studied. However, the existence of prophages in environmental *C. difficile* strains from a range of environmental sources is yet to be investigated.

In the present study, WGS revealed the distribution of various numbers of predicated prophages in all the isolates analysed. The most predominant intact prophages were phiCDHM1, phiCDHM19, phiMMP01, phiCD506, phiCD27, phiCD211, phiMMP03, phiC2, and phiMMP02, all of which are temperate *Myoviridae* or *Siphoviridae* belonging to the order of *Caudovirales*. One of the next steps in our work is the induction of predicated prophages from their host isolates and, subsequently, confirming the phage infection experimentally. To the best of our knowledge, this is the first study identifying two newly discovered phiSM101- and phivB_CpeS-CP51-like *Clostridium* phages in *C. difficile* genomes, which are similar to other prophages found in the genomes of *C. difficile* W0003a and NT64 reference strains. The phages phivB_CpeS-CP51 and phiSM101 were initially recognised as temperate bacteriophages of *C. perfringens* strains [24,27], and the phivB_CpeS-CP51 was later reported in the genomes of *C. chauvoei* [28] and *C. septicum* [29]. Up to 12 distinct prophages have been identified in a single genome of *C. difficile* isolates, with intact prophages ranging from one to six per isolate. It has recently been observed that predicted prophages range from three to 19 prophages in a single genome of a *C. difficile* strain, while intact prophages number more than three per strain [30]. However, to our knowledge, this is the first study to investigate the diversity of prophages in the genomes of a large number of environmental *C. difficile* RT/ST strains. Intact prophages were identified in distinct ST strains, including ST11, ST8, ST3, ST109, and ST2, as well as in different RT strains, namely RT127, RT001, RT073, RT014, RT018, RT120, RT023, and RT015. These findings indicate a clear correlation between prophage types and ST/RT. For instance, the predominant prophages identified in ST11 strains from clade 5, including “hypervirulent” RT strains RT127, RT126, and RT078, were phiCD506, phiCDHM1, phiCDHM19, and phiCD27. Furthermore, it should be noted that the prophage phiCD506 was exclusively identified in ST11/RT127 strains. These findings indicate that these prophages present in the *C. difficile* genomes could confer functional immunity against the corresponding phages. In a recent study, diverse prophages were discovered within the ST37 and ST81 isolates, which included the predominant prophage phiCD506 [30]. The results of this analysis show that there are a variety of prophage carriage patterns within isolates of the same RT/ST.

However, it is common to observe between one and three prophages along with genomic “islands” harbouring phage-related genes. In the current study, different genome sizes of identified prophages, specifically phiCD211, phiCD38-2, and phiCD6356, are maintained as extrachromosomal plasmids in *C. difficile* genomes. It was also revealed for the first time that the prophages phiCD506, phiCDHM14, and phiCD111 also exist as independent plasmids in their *C. difficile* genomes. These prophages were represented by different STs, ranging from one to ten different STs. Recent studies have highlighted the occurrence of large phage genomes that exist as extrachromosomal DNA plasmids in *C. difficile* genomes [18,31,32]. The initial discovery of a large phage, with a genome of approximately 131 kb, was phiCD211/phiCDIF1296T [9,18,33]. This large phage has been identified in 5% of 2584 analysed *C. difficile* genomes across 21 different STs [18,31,33]. Other large phage genomes, namely phiCD5763, phiCD5774, and phiCD2955, have recently been noted in isolates of *C. difficile* genomes, with representation of seven distinct STs [32].

Extrachromosomal phage genomes may be challenging to distinguish from large plasmids that contain phage-related genes. Phages that form extrachromosomal DNA plasmids, such as phage P1, use ParA/ParB, partitioning homologous, to maintain their plasmids during the lysogeny cycle [9,34]. In the current investigation, phiCDHM14 and phiCD6356 prophages are maintained as independent plasmids and the *parA* gene was identified in some of them. However, the *parA* gene has also been reported in phages, such as phiCD6356 [35], phiCD38-2 [16], and phiSemix9P1 [36]. It is impossible to determine the prophage maintenance mode solely based on the presence or absence of the partition gene. Therefore, it is difficult to conclude the exact nature of these large “extrachromosomal plasmids” without assessing their inducibility and production of infectious particles.

Many *C. difficile* prophage genomes contain genes suspected to impact their hosts. For example, in some phiCDHM1, a third type of the *agr* locus encodes the Agr-QS system, consisting of three genes: *agrD*, *agrB*, and *agrC*. Subsequently, Agr QS genes, *agrC* and *agrD* were also found to exist in other prophages, such as phiMMP01, phiMMPO2, and phiMMP03. The Agr-QS system of *Staphylococcus aureus* is the most extensively studied in Gram-positive bacteria. It is encoded by a four-gene operon consisting of *agrD*, *agrB*, *agrC*, and *agrA* [37]. The phiCDHM1 was the initial *C. difficile* phage to encode QS genes [17]. As phiCDHM1 lacks the *agrA* homolog, the precise function of this gene in QS in *C. difficile* requires further investigation. In the current study, another predicted accessory function is the Abi systems, which was identified in some of both phiC2 and phiMMP01. Notably, Abi genes were exclusively found in phiC2 [38]. The presence of the Abi genes in the genomes of phiC2 and phiMMP01 suggests that they may inhibit secondary phage infection or modulate phage replication and could contribute to the low frequencies reported for free phage isolation. A gene encoding a putative spore protease YyaC was also identified in 19 out of 30 phiCD211 prophages. Recently, the YyaC gene has been detected in phiCD211 of *C. difficile* genomes [18]. Spore germination is a crucial stage in the life cycle of *C. difficile* as it is necessary for vegetative growth, colonisation, and toxin production [39]. The DOC family protein was detected in 50% of phiCD211 prophages analysed in this study. Nevertheless, this protein was also displayed in some of the 149 phiCD211 prophages among the 2584 analysed *C. difficile* genomes [18]. DOC protein is also part of a toxin–antitoxin module Phd (prevents host death)-DOC from prophage P1, and a loss of the prophage should lead to host death [40].

Phage TerL sequences have previously been used to reconstruct the evolutionary relationships among different phages [9,41], indicating their potential to predict DNA packaging strategies based on the amino acid sequences of TerL proteins. Also, the phylogenetic relationship was constructed based on the amino acid sequences of LysM proteins, indicating that prophages might be shared with identical host cell lysis. The phylogenetic analysis of the *terL* gene from certain prophages, including phiCDHM14, phiMMPO4, and phiCD506, revealed clustering based on TerL sequences, which was consistent with the LysM phylogenetic tree analysis. In contrast, the phiCDHM19 phage forms a cluster with phiCDHM1 and phiMMP03. This cluster is not compatible with the LysM tree analysis, proving that these phages, with closely related TerL proteins, share comparable DNA packaging strategies. It demonstrates that these prophages could have comparable host cell lysis due to sharing the same evolutionary history of the phage genomes despite varying DNA packaging approaches.

The genome annotation revealed notable features of two newly identified phivB_CpeS-CP51- and phiSM101-like *Clostridium* phages and encode predicted proteins involved in DNA packaging, head morphogenesis, tail assembly and structure, DNA replication and recombination, and lysogenic control. The G+C content is similar to that identified in *C. difficile* NT64 and W0003a strains, as well as the published *C. perfringens* phage genomes of phivB_CpeS-CP51 [27] and phiSM101 [24]. The genome lengths of phivB_CpeS-CP51- and phiSM101-like *Clostridium* phages were 30.7 kb and 41.5 kb, respectively, whereas the genomes of *C. perfringens* phages were approximately 39 kb in length [24,27]. Notably, the genome of both newly discovered prophages did not have CRISPR-associated Cas systems, leading to the hypothesis that these prophages may not offer any functional immunity against the corresponding phages due to the lack of spacers.

The CRISPR-Cas systems found in the majority of prokaryotes offer adaptive protection against MGEs, such as phages and plasmids [42,43,44]. In addition, CRISPR arrays and CRISPR-associated (Cas) systems encode CRISPR RNAs (crRNAs) and Cas proteins, respectively, which play important roles in the adaptive immunity system in prokaryotes [43]. Numerous CRISPR arrays, like 505/1042 (48%) and 313/372 (84%), were homologous to all predicted and intact prophages, respectively, that were identified among the *C. difficile* prophage genomes in this study, further supporting that the CRISPR arrays in these predicted prophages could be proactive and preventative after subsequent infection by corresponding phages. In this study, all predicted prophages contain multiple CRISPR arrays, each with a varying number of spacers and different DR sequences and respective families present in the genomes of different RT/ST strains. This indicates a clear correlation between DR sequences, RT/ST and prophage types. The location of CRISPR arrays in phage genomes indicates the possibility of their transfer through transduction via HGT by lysogenic phages, thereby conferring protection against phage infection to new lysogenic phages [17,43]. Boudry et al. [21] reported a good correlation between the presence or absence of CRISPR spacers in *C. difficile* genomes and their susceptibility to phage infection. The authors noted that this method was a reliable predictor of susceptibility. In another study, multiple matches between CRISPR spacers were detected in the genomes of 31 *C. difficile* phages and prophages [45]. The Type-I CRISPR-Cas system was identified with the *cas3a* gene in the genomes of incomplete and questionable prophages. In this study, a new Type-IIIA CRISPR-associated *csm2* gene was found in the phiCDHM14 genome. Csm2, Csm3, and Csm5 could be required for crRNA maturation [46]. Recently, it has been shown that *S. aureus* MSHR1132, 08BA02716, *S. epidermidis* R62a and *S. capitis* CR01 have the type III-A CRISPR-Cas system, which contains the Cas proteins, Cas1, Cas2, Csm1, Csm2, Csm3, Csm4, Csm5, Csm6, and Cas6 [46,47,48]. The CDKM15 phage possesses a CRISPR array that could potentially target sequences of various *C. difficile* strains; however, no Cas genes were found in this phage [41]. Further research into the anti-phage defences of *C. difficile* RT strains will be crucial in predicting the effectiveness of phage-based therapy.

## 4. Materials and Methods

### 4.1. Whole Genome Sequencing and Data Analysis

Different strains of *C. difficile* were recovered from diverse environmental sources, as previously described [49]. After identification and phenotypic characterisation, 166 *C. difficile* isolates were subjected to WGS, and further phylogenetic and epidemiological characterisation revealed a clear relationship between those *C. difficile* strains [22]. MLST Sequence Types (STs) were extracted in accordance with the *C. difficile* MLST database of the PubMLST website (https://pubmlst.org/organisms/clostridioides-difficile, accessed on 15 November 2022). All sequenced contigs were annotated using the RAST web server (the rapid annotation using subsystem technology) version 2.0 (https://rast.nmpdr.org/, accessed on 15 November 2022). All contig sequences were deposited to NCBI GenBank under BioProject No. PRJNA1011814 [22]. The accession numbers for the sequences of contigs are listed in Table S1.

Prophages were predicated in 166 previously sequenced *C. difficile* genomes using the PHASTER (PHAge Search Tool–Enhanced Release) web server (https://phaster.ca, accessed on 14 August 2023). The intact, questionable, and incomplete prophage sequences were defined by score values of >90, 70 to 90, and <70, respectively [23]. Only hits with intact phages were considered for further analysis. CRISPR arrays and Cas-systems were identified in the intact prophage regions using CRISPRCasFinder [26].

### 4.2. Phylogenetic Analysis

A phylogenetic tree was generated using maximum likelihood (ML) analysis of the LysM proteins of 15 representative complete prophages at the amino acid level to determine whether prophage genes involved in the host cell lysis share the same evolutionary history as the phage genomes in general. Verified amino acid sequences of LysM proteins were aligned using MUSCLE in MEGA software (version 11) (http://www.megasoftware.net, accessed on 19 September 2023), which was then used to generate a ML tree.

To predict the DNA packaging strategies of 15 analysed prophages, including the newly identified phiSM101- and phivB_CpeS-CP51-like *Clostridium* phages, the sequences of the TerL gene were used. From 15 analysed prophages, along with two references for phiSM101- and phivB_CpeS-CP51-like *Clostridium* phages, which were identified using the PHASTER web server in the genomes of *C. difficile* NT64 and W0003a strains (GenBank accession no. CP101707.1 and CP025047.1, respectively), as well as two previously sequenced *C. perfringens* phage genomes, phiSM101 and phivB_CpeS-CP51, under accession numbers NC_021325.1 and NC_008265.1, respectively, were aligned at the amino acid level using MUSCLE in MEGA. The ML tree was performed using MEGA.

### 4.3. Features of Newly Identified Prophages

Protein coding genes were predicted in the genome regions of newly discovered phivB_CpeS-CP51- and phiSM101-like *Clostridium* phages using the RAST web server, Glimmer 3 [50], Geneious prime version 2023.2.1 (https://www.geneious.com, accessed on 10 October 2023), BV-BRC version 3.31.12 (https://www.bv-brc.org, accessed on 11 October 2023) [51], PHASTER web server [23], and BLASTn in NCBI database (https://blast.ncbi.nlm.nih.gov/Blast.cgi, accessed on 11 October 2023). The genome maps of newly identified prophages were generated using Geneious prime.

## 5. Conclusions

Many different prophage types were found in the sequenced genomes of environmental *C. difficile* isolates of different origins. By molecular genetics and bioinformatic analysis, the diversity of prophages identified in environmental *C. difficile* genomes will provide new opportunities to better understand their role in the evolution, physiology and virulence of this important human and zoonotic pathogen and to provide a basis for the development of phage-based therapy to treat CDI. The majority of the predicted prophages in the genomes of *C. difficile* strains harboured CRISPR arrays, indicating that prophages could play an important role in the defence mechanism of *C. difficile*. The induction of these identified prophages and their experimental confirmation of infection against various *C. difficile* RT strains with the presence or absence of CRISPR arrays are necessary and should be further investigated.

## Figures and Tables

**Table 1:** Characterisation of phage-unrelated genes in intact prophages identified in *C. difficile* genomes.

Target Genes	No. of Prophages (%)							
	phiMMP01	phiMMP02	phiMMP03	phiC2	phiCD211	phiCDHM1	phiCDHM14	phiCD6356
Agr-QS systems	*agrC*, *agrD* (1/39, 3%)	*agrC*, *agrD* (1/11, 9%)	*AgrC*, *agrD* (1/25, 4%)	-	-	*agrC*, *agrB*, *agrD* (4/78, 5%)	-	-
Spore protease YyaC	-	-	-	-	19/30 (63%)	-	-	-
Abi systems	19/39 (49%)	-	-	6/21 (29%)	-	-	-	-
Death-on-curing (DOC) protein	-	-	-	-	15/30 (50%)	-	-	-
*parA*	-	-	-	-	-	-	1/1 (100%)	2/6 (33%)

**Table 2:** CRISPR arrays in the intact prophages identified in *C. difficile* genomes.

RT/ST ^1^	Prophages	Family	CRISPR No.	Spacer No.	DR No.	DR ID ^2^
RT005/ST6	phiMMP01	*Myoviridae*	2	14, 3	21, 2	R7326, R8849
	phiMMP01		4	6, 17, 3, 2	19, 0	R7360, UN
	phiCDHM19		1	1	0	UN
RT090/ST1073	phiMMP01	*Myoviridae*	2	2	9	R1411
RT011/ST36	phiC2	*Myoviridae*	3	2, 17, 3, 19	21, 2	R7326, R8849
UC/ST2	phiMMP01	*Myoviridae*	4	2, 3, 17, 6	0	UN
	phiMMP01		2	3, 14	0, 9	UN, R1411
	phiCD6356	*Siphoviridae*	1	1	0	UN
RT020/ST2	phiC2	*Myoviridae*	2	5, 8	1	R7327, R6417
	phiMMP01		3	5, 4, 12, 6, 14	21, 0, 19, 9	R7326, R7360, R1411, UN
RT070/ST55	phiMMP02	*Myoviridae*	1, 2	3, 4, 6	2, 9	R1411, R3412
RT159/ST8	phiCD211	*Siphoviridae*	1	1	0	UN
	phiC2	*Myoviridae*	2	4, 13	0	UN
RT015/ST44	phiCDKM15	*Myoviridae*	2	6	0	UN
	phiMMP03		2	7, 10	0, 1	R7327, UN
	phiCDHM19		1	1	0	UN
	phiMMP01		1	3	0	UN
RTUC/ST254	phiMMP03	*Myoviridae*	1, 2	5	0, 19	R7360, UN
	phiMMP01		4	5, 7, 10, 14	0, 9	R1411, UN
	phiCDHM1		2	5	0, 1	R6417, UN
RT010/ST15	phiMMP01	*Myoviridae*	2	8, 13	21	R7326
RT140/ST26	phiMMP01	*Myoviridae*	2	2, 13, 14	1, 21	R7326, R7327
RT140/ST515	phiMMP01	*Myoviridae*	2	2, 14	21	R7326
RT023/ST5	phiMMP02	*Myoviridae*	2	3, 7	0, 9, 21	R7326, R1411, UN
	phiCDHM1		1	3, 14	9, 21	R1411, R7326
	phiCD505		1	13	21	R7326
RT014/ST14	phiMMP01	*Myoviridae*	3	4, 6, 14	0, 9	R1411, UN
	phiCDKM15		2	3, 5	0, 9	R1411, UN
RT014/ST2	phiMMP03	*Myoviridae*	1, 2	5, 7, 8	0, 1	R7327, R6417, UN
	phiMMP01		3	4, 5, 6, 11, 12	0, 9, 19, 21	R7326, R7360, R1411, UN
RT014/ST13	phiMMP01	*Myoviridae*	3	4, 6, 14	0, 19, 21	R7326, R7360, UN
RT014/ST49	phiCDKM15	*Myoviridae*	1	6	0	UN
	phiMMP01		3	4, 6, 14	0, 19, 21	R7326, R7360, UN
RT018/ST17	phiCDHM1	*Myoviridae*	4	3, 5, 6	0, 2, 9, 21	R1411, R7326, R8849, UN
	phiMMP02		1	17	9	R1411
	phiMMP01		1	5	0	UN
RT001/ST3	phiC2	*Myoviridae*	4	1, 3, 7, 13	0, 9	R1411, UN
	phiCDHM1		3, 4	2, 3, 4, 5, 6	0, 1, 9, 19, 21	R7360, R7326, R1411, R8402, UN
	phiCDKM9		1	2	0	UN
	phiCDHM19		1	1	0	UN
RTUC/ST821	phiMMP03	*Myoviridae*	2	8, 11	19	R7360
RTUC/ST917	phiMMP01	*Myoviridae*	2	1, 5	0	UN
RT126/ST11	phiCDHM19	*Myoviridae*	1	3	9, 21	R7326
	phiCD27		1	4	0	UN
RT031/ST26	phiMMP03	*Myoviridae*	2	4, 5	0	UN
RT017/ST37	phiMMP01	*Myoviridae*	2	3, 4	0, 1	R8649, UN
	phiCDHM19		1	1	0	UN
RT002/ST8	phiMMP01	*Myoviridae*	2	4	0, 19	R7360, UN
	phiMMP03		1	1	0	UN
RT127/ST11	phiCD27	*Myoviridae*	1	4	0	UN
	phiCDHM19		1	3	9, 21	R1411, R7326
	phiMMP02		1	4	0	UN
RTUC/ST11	phiCDHM1	*Myoviridae*	1	5	0	UN
	phiCDKM15		1	8	0	UN
	phiCD27		1	4, 8	0	UN
RTUC/ST11	phiCDHM19	*Myoviridae*	1	3	21	UN
RT095/ST2	phiMMP01	*Myoviridae*	3	4, 6, 14	0, 9	R1411, UN
RT077/ST13	phiMMP01	*Myoviridae*	3	4, 6, 14	0, 9, 21	R7326, R7360, UN
RT120/ST4	phiMMP01	*Myoviridae*	1	5	0	UN
	phiCDKM9		1	5	0	UN
RT328/ST35	phiMMP03	*Myoviridae*	2	8, 9	0, 9	R1411, UN
	phiC2		2	8, 9	0, 9	R1411, UN
RT103/ST53	phiCD27	*Myoviridae*	3	1, 3, 5	0	UN
	phiMMP01		2	5, 6	21	R7326
RT073/ST109	phiC2	*Myoviridae*	3	5, 9	0, 9, 21	R7326, R1411, UN
	phiCDHM1		1	1	0	UN
RT085/ST39	phiC2	*Myoviridae*	3	5, 8, 12	0, 21	R7326, UN

^1^ UC: unclassified; ^2^ UN: unknown.

## Data Availability

Data are presented in the article and Supplementary Materials.

## Supplementary Materials

The following supporting information can be downloaded at: https://www.mdpi.com/article/10.3390/ijms25010002/s1.
