# Supplementary material for: Prophage Carriage and Genetic Diversity within Environmental Isolates of Clostridioides difficile
Source: Int J Mol Sci. 2023 Dec 19;25(1):2. doi: 10.3390/ijms25010002 (PMC10778935; doi:10.3390/ijms25010002)
Supplement: Supplementary file 1 [file ijms-25-00002-s001.zip › Table S5.pdf]

Table S5. Cas systems in identified prophage genomes of environmental *C. difficile* isolates.

Strain name	Region/CRISPR ID	Completeness / prophage type	Cas-type/subtype	Gene status	System	Start	End	Strand
C. difficile RSS39	2_2	Incomplete	cas3a_TypeI	Forbidden	CAS	842	1414	+
C. difficile CF76	1_35	Questionable	cas3a_TypeI	Forbidden	CAS	23934	24875	-
C. difficile RS147	7_20	Incomplete	cas3a_TypeI	Forbidden	CAS	18067	18067	-
C. difficile RS150	3_20	Incomplete	cas3a_TypeI	Forbidden	CAS	18067	18067	-
C. difficile ARC182	8_44	Intact (phiCDHM14)	csm2_TypeIIIA	Mandatory	CAS-TypeIIIA	27400	27660	-
